# Child Crying in Utero

**Published:** 1876-06

**Authors:** W. H. Dean

**Affiliations:** Woodstock, Ga.


### CHILD CRYING IN UTERO.

*Editors Atlanta Medical and Surgical Journal:*

Gentlemen—I was called on the 7th inst. to see a woman in labor with her fourth child. The membranes ruptured about twenty minutes before I arrived, and a large quantity of liquor amnii was discharged. Made an examination immediately; found the cord prolapsed and the head above the superior strait. The woman got up to have her bed arranged, and while up the child in utero commenced crying vigorously, so as to be heard all over the room, and if persons had been listening, could have been heard all through the house.

This being the first case witnessed in a practice of about thirty years, I concluded to report it.

W. H. Dean, M.D.

*Woodstock, Ga., April* 18, 1876.

(It is to be regretted that Dr. Dean did not give a more detailed report of his case. The length of time that elapsed after the cries of the child were heard and its birth, and the mode adopted to verify that the noise heard emanated from the child, would be of interest. We hope the Doctor will furnish for our next issue a more complete report of this very interesting case.—Eds.)

